# Evaluation of the Surface Topography and Deformation of Vertical Thin-Wall Milled Samples from the Nickel Alloy Inconel 625

**DOI:** 10.3390/ma17020295

**Published:** 2024-01-07

**Authors:** Szymon Kurpiel, Krzysztof Zagórski, Jacek Cieślik, Krzysztof Skrzypkowski, Amandyk Tuleshov

**Affiliations:** 1 Faculty of Mechanical Engineering and Robotics, AGH University of Krakow, Mickiewicza 30 Av., 30-059 Krakow, Poland; 2 Faculty of Civil Engineering and Resource Management, AGH University of Krakow, Mickiewicza 30 Av., 30-059 Krakow, Poland; 3 Joldasbekov Institute of Mechanics and Engineering, Kurmagazy Str. 29, Almaty 050010, Kazakhstan

**Keywords:** vertical thin-walled sample, milling, nickel alloy, Inconel 625, surface topography, waviness, roughness, deviation, deformation, 3D optical scanner

## Abstract

During the production of components, manufacturers of structures are obliged to meet certain requirements and ensure appropriate quality characteristics. It is especially important during the manufacturing of thin-walled structures, which are subject to many errors during machining due to the reduced rigidity of the products, including the deformation of thin walls, which may be the result of the vibration of the system. The appearance of vibrations reduces the quality of the machined surface affecting the increase in the values of surface topography parameters—waviness and roughness. Thin-wall structures—titanium or nickel alloy, among others—play a key role in the aerospace industry, which constantly strives to reduce the weight of the entire structure while meeting requirements. The present work focuses on the evaluation of the parameters of surface topography, dimensional and shape accuracy during the milling of nickel alloy Inconel 625 samples containing a thin wall in a vertical orientation. The experiment was conducted under controlled cutting conditions using a constant material removal rate. As part of the surface topography section, the distribution of waviness, Wa and Wz, and roughness, Ra and Rz, was determined in selected measurement areas in the direction parallel to the direction of the feed motion. Dimensional deviations, measured with a 3D optical scanner, were determined in selected cross sections in the direction perpendicular and parallel to the bottom of the sample presenting the deflection of the thin-walled structure. The results provide information that the used parameter sets affect the measured quantities to varying degrees.

## 1. Introduction

Ensuring the sufficient quality of finished products used for various structures is one of the requirements of their manufacturers. Such an endeavor increases the durability and operation of mating components. The aim is to obtain whatsoever more accurate components to meet the assumptions made at the design stage [1]. The use of higher accuracies forces manufacturers to use narrower tolerances [2,3,4]. Such action forces parallel development not only in metrology but also in the manufacturing of machine components and the development of the machining center [5,6,7,8].

In the field of metrology, the development of measurement methods is observed [9,10,11,12]. In addition to the use of classical measurement methods, such as contact measurements using a coordinate measuring machine, interest in optical methods is evident [13,14]. The greatest advantages of optical methods are shorter measurement times and the possibility of the dimensional inspection of the entire product, rather than in a point or linear manner as in the case of CMM [15,16,17]. One of the newer measuring devices using the optical method is the 3D optical scanner (GOM). It is most commonly used in many industries to inspect components after the manufacturing process [18,19]. The possibility of using an optical scanner to measure thin-walled components with small dimensions was presented in study [1], while in study [15], we presented the correlation of results obtained using a 3D optical scanner and a coordinate measuring machine. In study [15], samples with thin walls in a horizontal orientation were tested, for which a maximum measurement discrepancy of 8% was obtained between the methods used.

The presented studies do not focus on the study of deviations in thin-walled structures from nickel alloys. One can find articles containing dimensional accuracy results for thin-walled samples in the vertical orientation from titanium alloy, for which the maximum deviation values are as follows: Zha [20] showed deviations equal to 0.21 mm, Yusop [21] equal to +0.18 mm, Hintze [22] and Gang [23] obtained similar deviation values equal to 0.1 mm, Polishetty [24] presented a maximum deviation equal to 0.18 mm (with a high roughness value—Ra_max_ = 3.109 μm). It is worth mentioning here that deviations can take positive (material allowance) or negative (material loss) values and are values of manufacturing error [25].

In the field of the manufacture of machine components, there is continuous development of machining machines, cutting tools and manufacturing methods [26,27]. These treatments ensure an increase in the quality characteristics of workpieces. On the other hand, design manufacturers strive to reduce manufacturing costs by looking for increasingly higher cutting parameters that will enable a reduction in machining time, all while meeting the assumed quality characteristics [28,29,30,31,32]. Such a vision makes it legitimate to conduct research in controlling the effect of output parameters on output quantities. 

Currently, there is interest in thin-walled structures [33] which, in terms of machining, are most often processed using milling. Milling machining enables the manufacture of products with complex shapes [1]. Their application to structures, including aerospace structures, makes it possible to reduce their weight, which consequently also affects operating costs. Parts containing thin-wall structures are obtained by removing up to 95% of the initial volume of the material, which significantly reduces the weight of the structure [34,35]. The desire to use thin-wall structures carries certain consequences. Machining such components is difficult, due to the reduced stiffness of the product [36]. Consequently, many undesirable phenomena occur during machining, as well as manufacturing errors [25,36,37]. The dynamic instabilities that occur during machining negatively affect the roughness of the finished product and cause an increase in tool and machining machine wear [3,38]. Additional factors affecting surface quality, including roughness, are cutting forces during machining and the way the workpiece is clamped [39,40].

The reduced stiffness of thin-walled products also affects the appearance of their deformations, which are a serious problem during machining because they contribute to geometric errors [36]. This phenomenon affects surface topography and is closely related to cutting conditions, including vibration and cutting forces [41,42,43,44,45,46,47]. Therefore, the causes of the anomalies are being sought, among other things, in the machining process and the possibility of eliminating them during future machining operations [47,48,49,50,51]. 

The purpose of this study is to check the influence of cutting tools and cutting strategies on the features of finished products determined by thin wall deviations and surface topography parameters—waviness and roughness—during the milling of thin wall nickel alloy samples in a vertical orientation. The results of this study will be compared in terms of the influence of the workpiece material with those presented in the study [1]. The strength of the present study is the use of a nickel alloy as a test material for thin-wall structures. Much of the presented works focuses on the study of aluminum alloy thin-walled components [36]. A few sparse works present the results of studies of thin-walled structures made of titanium alloy [20,21,22,23,24] by milling machining, while the occurrence of leading works using nickel alloy for thin-walled structures has not been observed. In paper [52], we presented the waveforms of vibration signals for thin-wall nickel alloy samples in a vertical orientation, which are the subject of the present study.

## 2. Materials and Methods

The research presented in this article is part of a larger study and complements the experiment presented in articles [1,52]. The conditions for machining the samples, as well as for measurement, were the same as in the case of publication [52]. This article will cite general information about the machining conditions and the measurement of the samples, as well as the adopted test methodology.

### 2.1. Experimental Setup and Cutting Conditions

**Figure 1:**
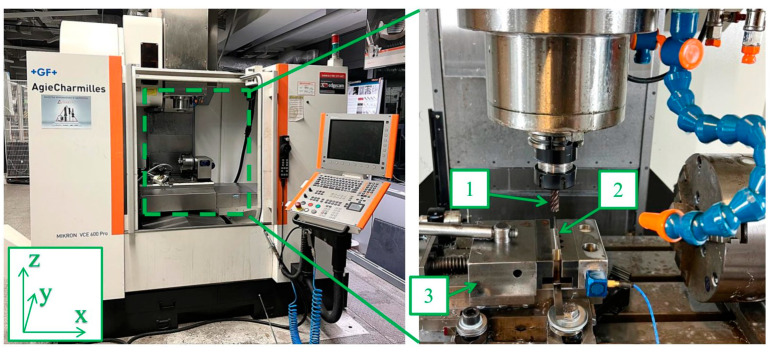
Machining center and instrumentation to conduct the experiment: 1—tool, 2—workpiece material, 3—vise.

The machining of the samples was carried out on a Mikron VCE 600 Pro machining center (Biel/Bienne, Switzerland) with control software iTNC 530 developed by Heideinhain (Traunreut, Germany). During milling, three types of ϕ10 diameter monolithic milling cutters supplied by Seco Tools were used: for general purposes JS554100E2R050.0Z4-SIRA (Tool 1), for high-performance machining JS754100E2C.0Z4A-HXT (Tool 2) and for high-speed machining JH730100D2R100.0Z7-HXT (Tool 3) [52,53,54,55]. The tools were mounted in a precision sleeve and then in an ER32 toolholder. The semi-finished part to be machined was mounted in a vice with a shank length of 10 mm from the base of the sample. According to the recommendations of the cutting tool manufacturer, SILUB MAX coolant was used during machining in the form of a mixture—15% oil emulsion and 85% water [52,56]. A graphical representation of the described test stand is shown in Figure 1.

**Figure 2:**
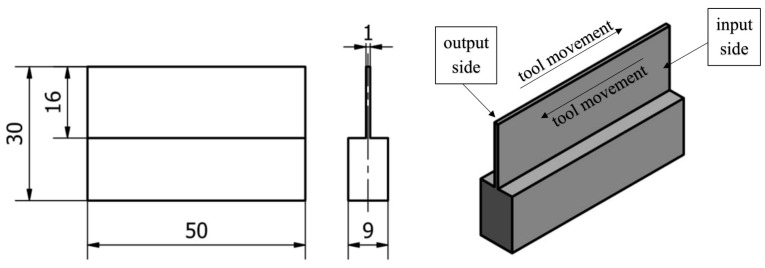
Documentation and description of the vertical thin-walled sample [52].

The assumed shape of the sample with a thin wall in a vertical orientation is shown in Figure 2. A thin wall is made along the entire length of the blank (50 mm), 16 mm high, while its thickness is 1 mm. The following designations of the machined surfaces are adopted: entry side—for the penultimate pass—and exit side—for the last pass. 

The material used in the experiment is Inconel 625 nickel alloy, which is a popular material in the aerospace industry, mainly as a material used in turbines [57,58]. The basic mechanical properties and chemical composition of this material are shown in Table 1 and Table 2.

A constant cutting speed of V_c_ = 40 m/min and a feed rate of V_f_ = 255 mm/min were adopted for machining the samples. Two different side milling approaches were used—face and cylindrical. The adopted depths of cut and radial depths under these strategies are shown in Table 3. The material removal rate depends on the feed rate, depth of cut and feed depth [1,15,52,60], so it can be seen from the values presented that it is constant at 2.03 cm^3^/min in the present experiment, which is the basis for comparing the results between samples.

**Figure 3:**
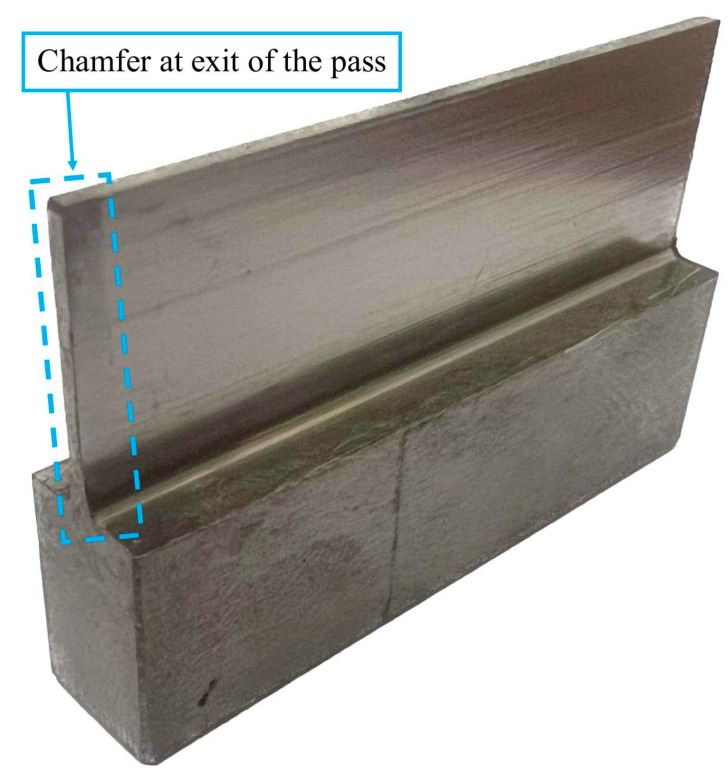
Representation of an example sample containing a thin wall in a vertical orientation with a post-treatment feature revealed.

Figure 3 shows a photo of an example sample prepared based on the described methodology.

### 2.2. Deformation Measurement

**Figure 4:**
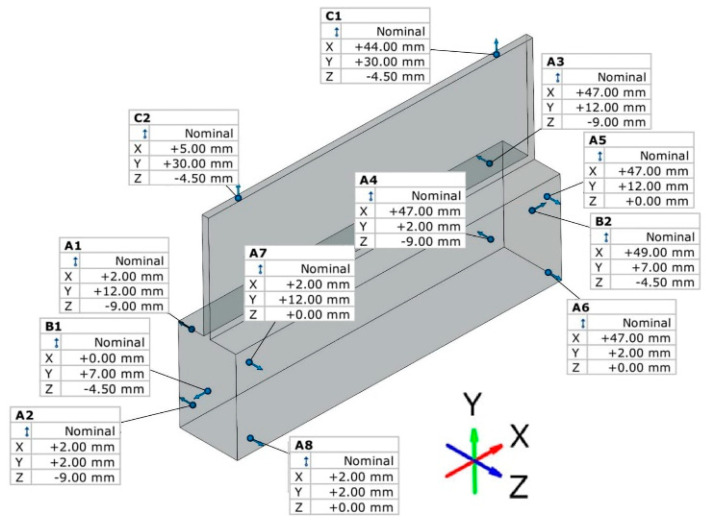
Method of basing during measurement with 3D optical scanner [52].

A series of measurements of thin wall deviations was carried out using a 3D optical scanner—Atos ScanBox 6130 designed by GOM (Braunschweig, Germany). The measurement of samples was carried out in a free state, i.e., the sample was not immobilized during the measurement relative to the adopted bases. The program and the measurement report were prepared in the dedicated software GOM Inspect 2020 (2020.0.4.135965). The adopted basing method for the preparation of the measurement report is shown in Figure 4.

**Figure 5:**
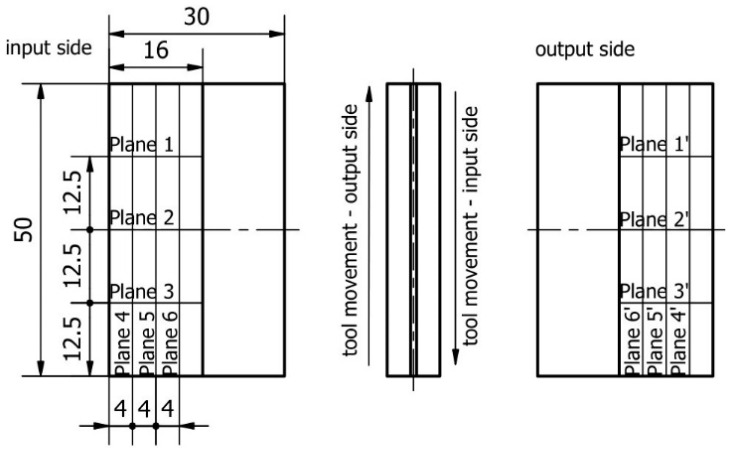
The designation of measuring planes for determining deviations [52].

Based on the obtained report, thin wall deflection diagrams were prepared in selected sections—in directions perpendicular and parallel to the bottom of the sample, as described and shown in Figure 5.

### 2.3. Surface Topography Measurement

**Figure 6:**
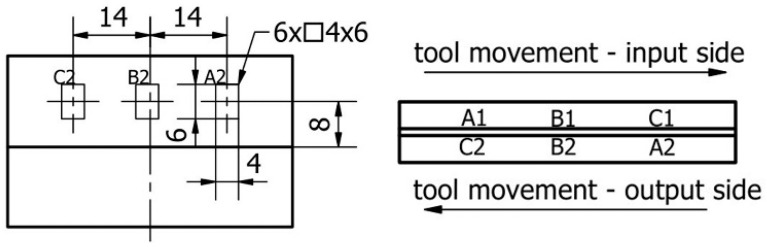
The designation of measuring areas for surface topography measurement [52].

Surface topography was measured using a Topo 01P v3D contact profilometer coupled to a MY120-AS sliding table. The recorded signal during reading with the measuring tip was transferred to dedicated software. As part of the measurement of the surface topography of the thin wall, 3 measuring areas were adopted for each of the machined sides. The measuring areas were determined in such a way that they presented the distribution of parameters in a direction parallel to the direction of feed motion. The description and designation of the adopted measuring areas are shown in Figure 6.

## 3. Results and Discussion

The deviation, roughness and waviness graphs presented within this chapter were prepared in MatLAB R2022b. The data used to prepare thin wall deflection plots and histograms of selected surface topography parameters were taken from measurement reports obtained during the test. Statistical analysis was carried out in Statistica v13 software using the option to determine basic descriptive statistics and box-plots.

It is worth mentioning that on the surface of the samples, the presence of a characteristic feature resembling a chamfer was observed (Figure 3). The feature occurred at the point where the tool left the material and occurred on both sides of the workpieces, both on the entry and exit sides of the tool from the material.

### 3.1. Deformation of the Thin Wall

**Figure 7:**
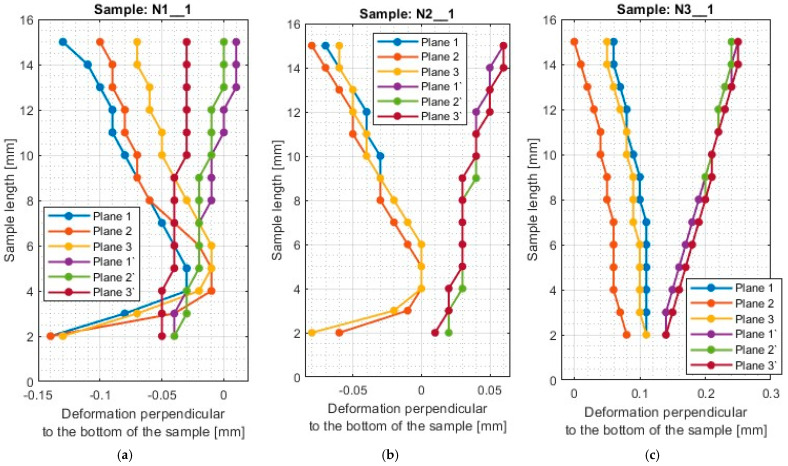
The deflection of the thin wall in the perpendicular direction to the bottom of the sample: (**a**) N1_1; (**b**) N2_1; (**c**) N3_1.

**Figure 8:**
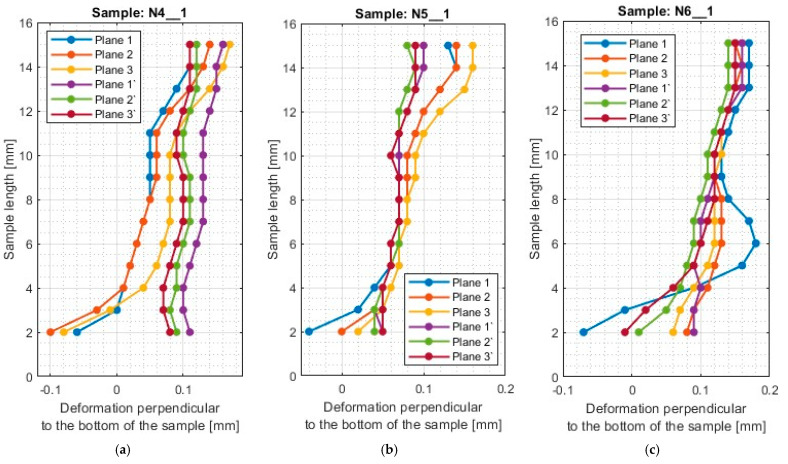
The deflection of the thin wall in the perpendicular direction to the bottom of the sample: (**a**) N4_1; (**b**) N5_1; (**c**) N6_1.

**Figure 9:**
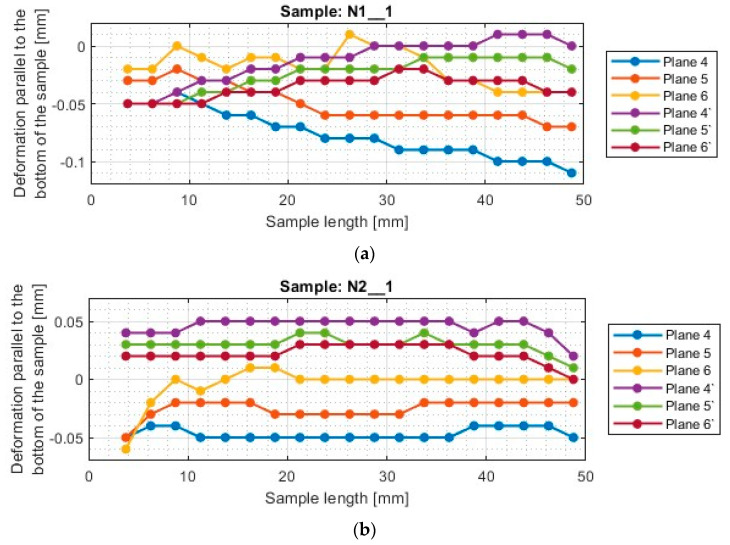

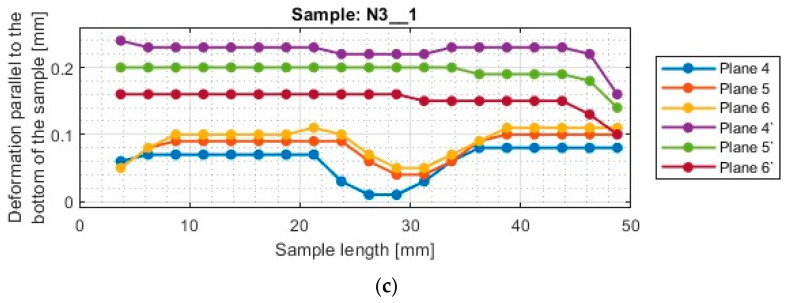
The deflection of the thin wall in the parallel direction to the bottom of the sample: (**a**) N1_1; (**b**) N2_1; (**c**) N3_1.

**Figure 10:**
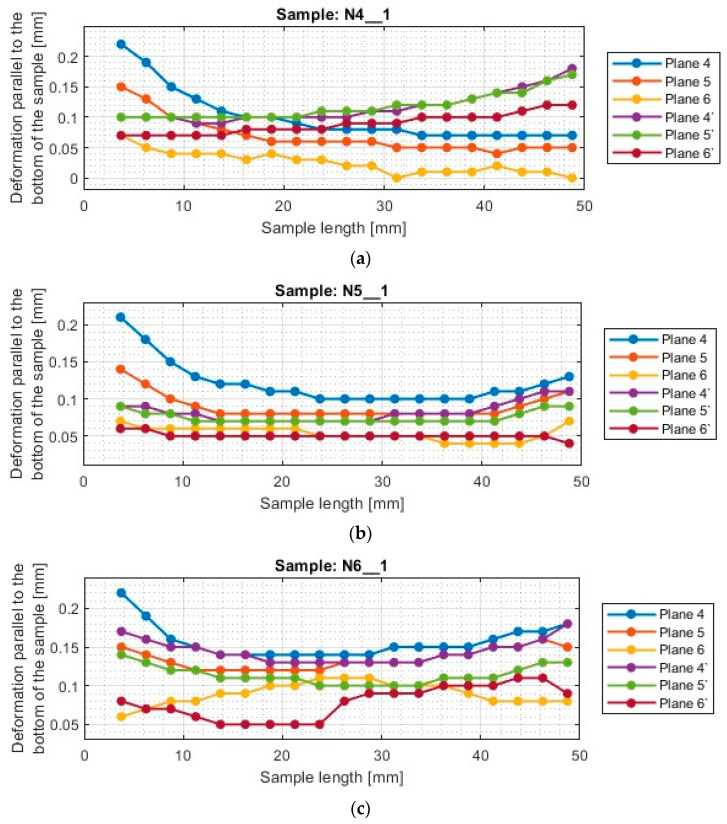
The deflection of the thin wall in the parallel direction to the bottom of the sample: (**a**) N4_1; (**b**) N5_1; (**c**) N6_1.

**Figure A1:**
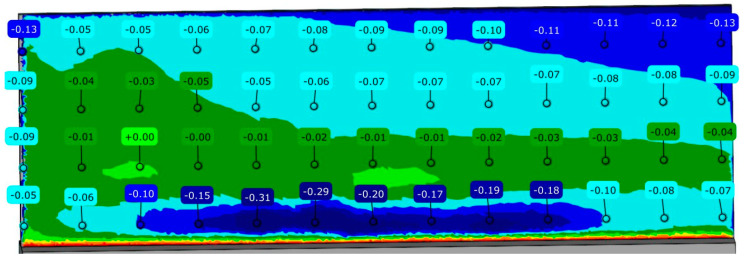
Color map for sample N1_1—input side.

**Figure A2:**
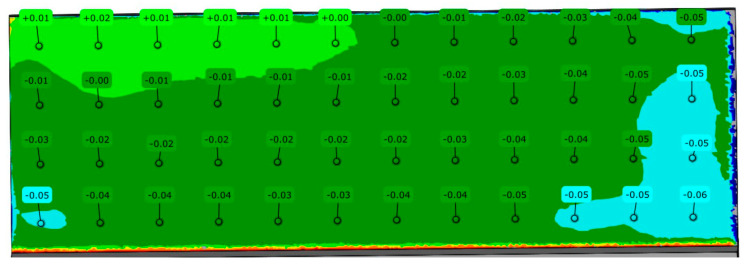
Color map for sample N1_1—output side.

**Figure A3:**
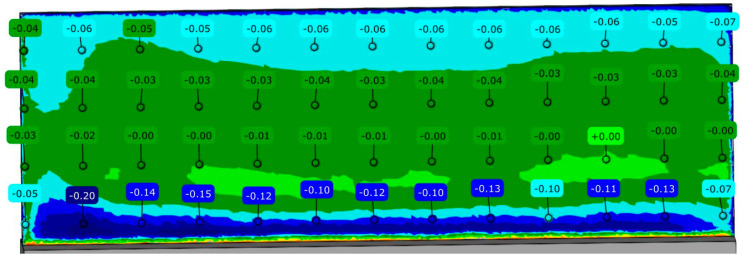
Color map for sample N2_1—input side.

**Figure A4:**
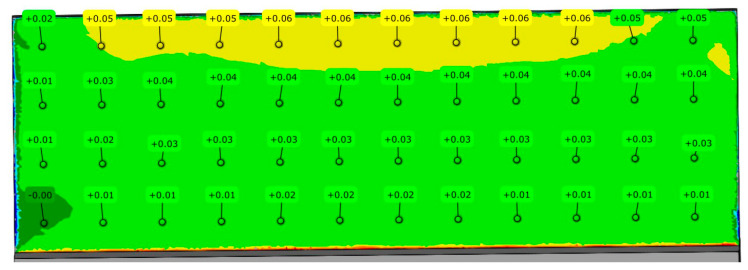
Color map for sample N2_1—output side.

**Figure A5:**
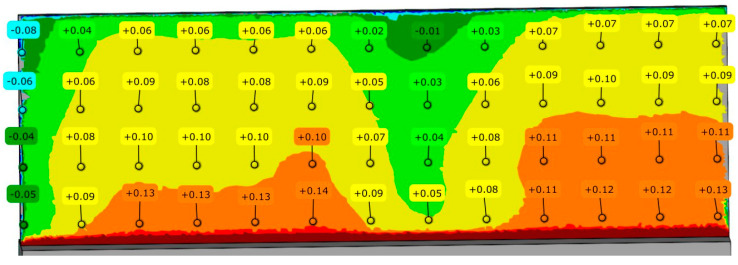
Color map for sample N3_1—input side.

**Figure A6:**
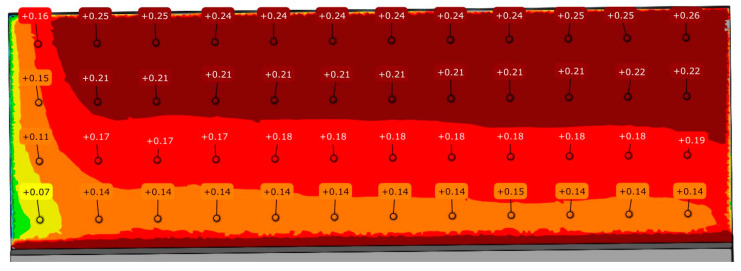
Color map for sample N3_1—output side.

**Figure A7:**
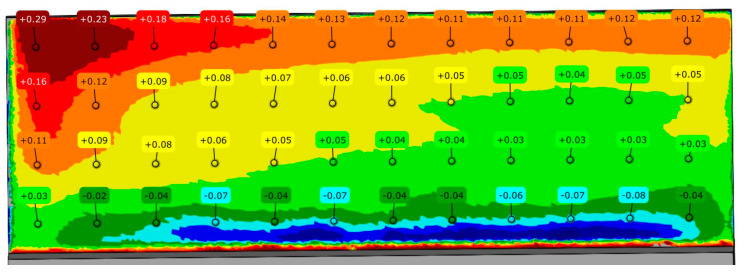
Color map for sample N4_1—input side.

**Figure A8:**
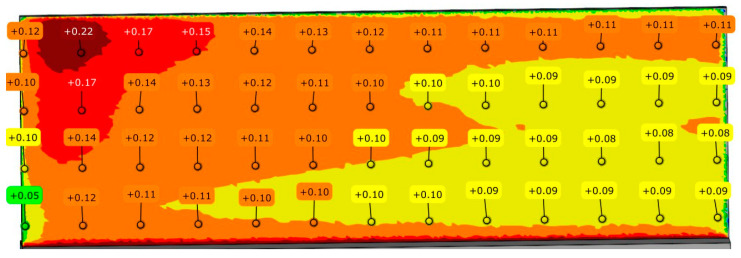
Color map for sample N4_1—output side.

**Figure A9:**
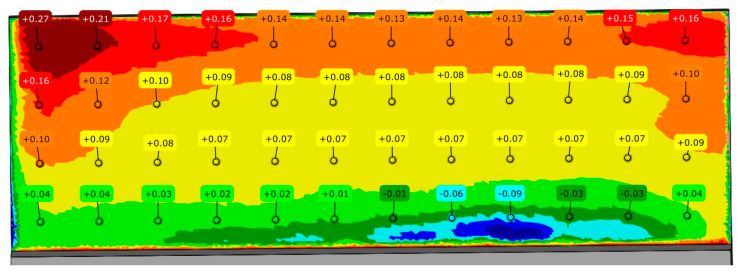
Color map for sample N5_1—input side.

**Figure A10:**
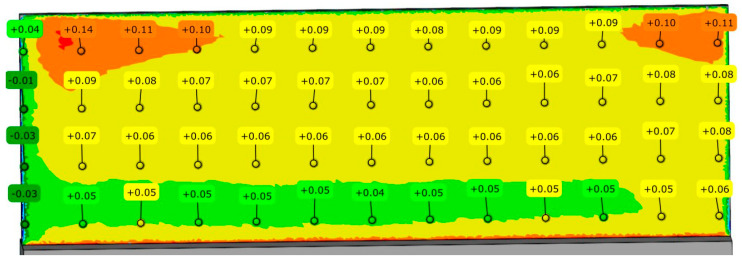
Color map for sample N5_1—output side.

**Figure A11:**
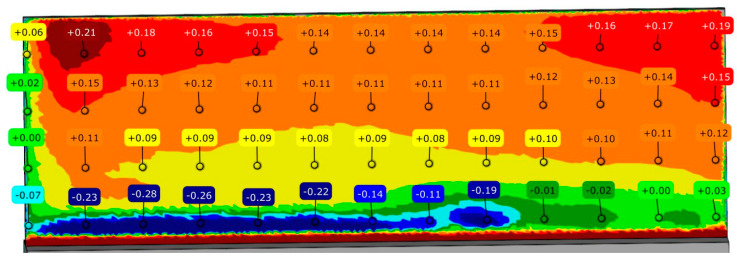
Color map for sample N6_1—input side.

**Figure A12:**
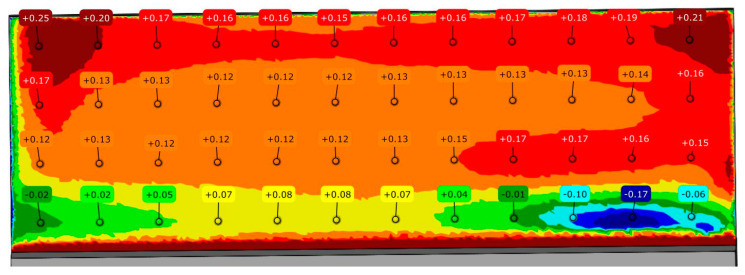
Color map for sample N6_1—output side.

A typical result in the report after measurement with a 3D optical scanner is a color map of the measured surface. In Appendix A, Figure A1, Figure A2, Figure A3, Figure A4, Figure A5, Figure A6, Figure A7, Figure A8, Figure A9, Figure A10, Figure A11 and Figure A12 show the deviations of the thin wall in the form of color maps, for both machined sides (input and output). Graphs of vertical thin-wall deviations in selected planes for nickel alloy samples in the direction perpendicular to the bottom of the sample are shown in Figure 7 and Figure 8 and the direction parallel to the bottom of the sample in Figure 9 and Figure 10. The results of the analyzed samples are presented graphically as a plot of thin-wall deviations versus length. The sign in front of the deviation value was consistently adopted as in the color charts in the measurement report. The placed sign only indicates the direction of the deviation. A negative sign indicates material loss, while a positive sign indicates material allowance. When reading the graphs, note that the cutter moved according to the direction of the *y*-axis (of the machine coordinate system, see Figure 1) on the exit side and opposite to it on the entry side. Therefore, for planes 4’–6’, located on the exit side of the tool from the material, the machining of the sample starts at 0 mm and the cutter moves up to 50 mm. For planes 4–6, which are on the tool entry side of the material, the machining of the sample starts at 50 mm and the cutter moves to 0 mm.

For the plots of thin wall deviations for the nickel alloy samples shown in Figure 7 and Figure 8, analogous to those for titanium alloy [1], some characteristic features appearing for each of the tools used (i.e., between N1_1 and N4_1, N2_1 and N5_1, N3_1 and N6_1) are evident.

Deviations for samples made with the general purpose tool using both strategies (N1_1 and N4_1) stabilize after a certain value of sample length is reached—for the charts analyzed, this value is 4 mm. At values below 4 mm, i.e., for values close to the bottom of the sample, the deviations are much larger. The smaller value required to stabilize the deviations—compared to titanium alloy [1]—is due to the higher stiffness of the workpiece material. Sample N1_1, like sample T1, has a scatter of deviation values at the base, then the value deviations are close to each other, after which an inverted cone shape is observed. For nickel alloy and titanium alloy samples [1] made with the tool for general purposes using a side face milling (T1 and N1_1), the surface on the entry side is characterized by a near-vertical graph shape. For sample N1_1, negative deviations are observed on the exit side, so the thin wall has a thickness less than assumed (less than 1 mm). Sample N4_1, similar to T3 (made under the same conditions) [1], shows a scattering of values at the base of the thin wall and then the graphs of deviations in different planes assume a similar character with similar values. In the case of this sample, the deviations presented are positive, so the thin wall has a greater thickness than the assumed value of 1 mm. Unlike the other samples, the thickness of the thin wall along the entire length of sample N4_1 is approximately constant.

The scatter of deviations at the base of the thin wall is also observed for the samples made with the tool for high-performance machining (N2_1 and N5_1), but it quickly stabilizes—as soon as the length of the sample reaches about 3 mm. The application of the side milling strategy (N2_1), shows that the thickness of the sample above its length equal to 3 mm is close to the assumed value (1 mm). However, it is observed that the thin wall is inclined to the exit side. The situation is different for cylindrical side milling (N5_1), in which the sample assumes the shape of an inverted cone, i.e., the thickness at the base of the thin wall is close to the assumed value of 1 mm and increases moving farther from the base. This is due to the deformation of the thin wall to the opposite side by the pressure of the tool on the machined surface during successive machining passes. Similar trends in the shape of the graph were observed for the counterparts of the titanium alloy samples (T2 and T4—made under the same conditions) [1]. Comparing the deviations for samples N2_1 and N5_1 with each other, smaller values by about half were obtained when using side face milling (N2_1).

The presented deviation graphs for sample N3_1, obtained during machining with the tool for high-speed machining using side face milling, are slightly different than was presented for the other nickel alloy samples. The graphs have a very regular shape—they are approximately straight lines oriented at corresponding angles. The graph—between the machined sides—assumes a shape that is close to symmetrical, which means that the sample had an almost constant thin wall thickness but skewed to the output side (due to the presence of positive deviations). A slightly different trend occurs for sample N6_1, made with the same cutter, but with the opposite strategy (cylindrical side milling). For sample N6_1, as for samples N4_1 and N5_1 obtained with the same strategy, the thin wall is characterized by a distribution of planes in a shape resembling an inverted cone. This means that the lowest deviations were obtained at the base of the thin wall, then their values increased further away from the base. For plane 1, a material allowance of between 4 mm and 8 mm appears. Comparing globally the deviation plots of the samples made using side milling cylindrical milling (N4_1–N6_1), sample N6_1 has the smallest spread of values between planes.

Summarizing the results obtained in the direction perpendicular to the bottom of the sample, it can be reported that when using a material with a higher stiffness—nickel alloy—it was observed that approximately constant sample thickness is obtained during the application of lateral face milling (N1_1–N3_1), however, slanted to one side. On the deviation diagrams of the samples made using cylindrical side milling (N4_1–N6_1), the shape of an inverted cone appears, which presents the smallest deviation at the base of the thin wall (thickness close to the assumed equal to 1 mm) and moving away from the bottom of the sample, more and more deformation appears. It can be concluded that at each pass for this tool, the sample was deformed to the opposite side.

Based on the plots of thin wall deviations in the direction parallel to the bottom of the wall for nickel alloy samples shown in Figure 9 and Figure 10, one also observes the similar nature of the distribution of the plots in each strategy (between N1_1–N3_1 and between N4_1–N6_1). For samples made with the frontal side milling strategy (N1_1–N3_1), the tendency of the location of the individual measurement planes is analogous. This means that the deviations for the input side are located in the lower part of the graph, while those for the output side are located in the upper part of the graph. However, despite this, it is important to pay attention to the sign in front of the deviation, since its occurrence makes each sample slightly different in shape and character.

Sample N1_1 showed a scattering of deviation values at individual measurement points, the greater influence of which is observed for the input side. The values of deviations indicate that the thin wall—in the top view—takes the shape of a cone, the apex of which is directed towards the beginning of the sample. The deviations presented are negative, so the thickness of the thin wall—despite the shape deformation—is less than assumed, with its smallest thickness registered at the end of the sample length. This graph confirms that the surface on the input side is skewed relative to its base.

For samples N2_1 and N3_1, the loss of material at the exit of the tool from the sample is visible—on both sides (at the length of the sample equal to about 5 mm and 25 mm). Outside these areas, the graphs are fairly regular and flat. Sample N2_1 takes an approximately symmetrical shape concerning the *x*-axis, so the thickness of the thin wall was close to the assumed value, but uniformly inclined to the exit side.

The deviations presented for sample N3_1 took positive values, indicating that the thickness of the thin wall took on a larger value than the assumed 1 mm. Since the deviations on the graphs of planes 4–6 presented similar values between them, the thin wall is inclined to the output side. In the middle part of sample N3_1, on the input side, a half-circle deformation is visible, whose deviation value is close to zero.

The surfaces of the samples obtained after machining with the cylindrical side milling strategy (N4_1–N6_1) show positive deviations for both machined sides—both on the entry and exit sides. At the exit of the tool from the sample (on both machined sides), a significant increase in deviations is seen, with larger values observed further away from the bottom of the sample. Excluding the aforementioned deviations located at the tool exit, the others presented have a fairly regular course—close to horizontal. Therefore, it is concluded that the samples are characterized by a larger thin wall thickness (compared to the assumed value), with a relatively constant value of it—ignoring the areas of tool exit from the sample. For samples made using this strategy (N4_1–N6_1), similar values of deviations between tools are observed.

### 3.2. Surface Topography

**Figure 11:**
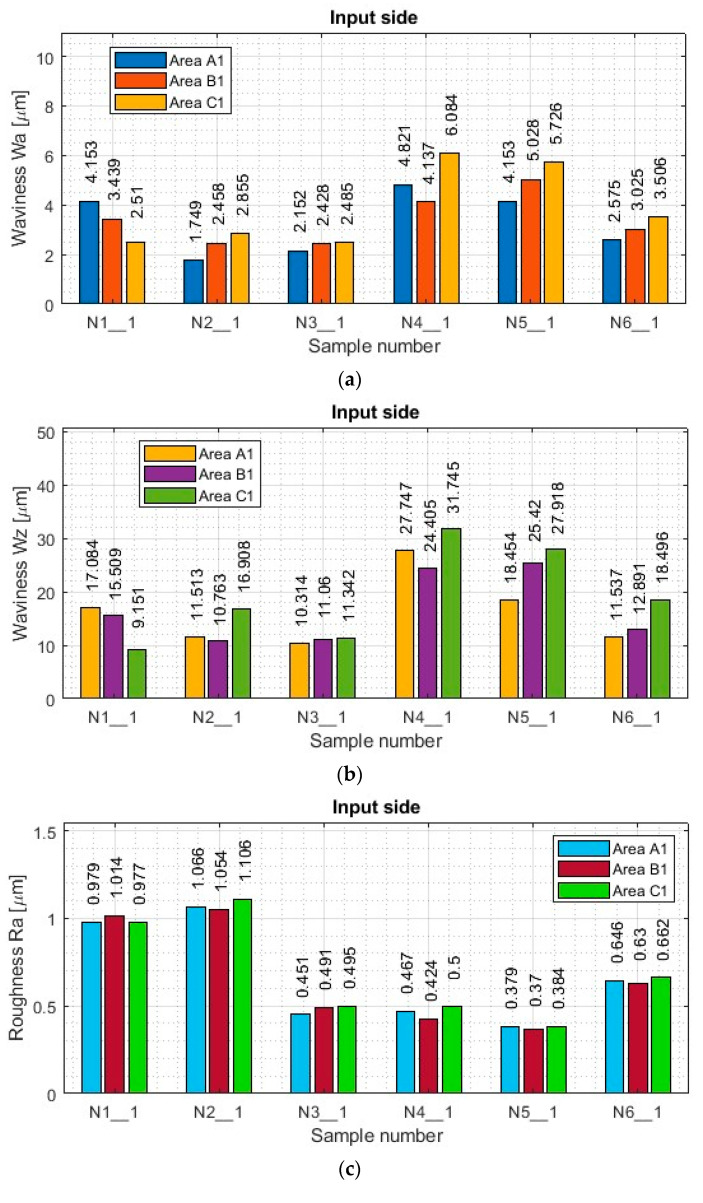

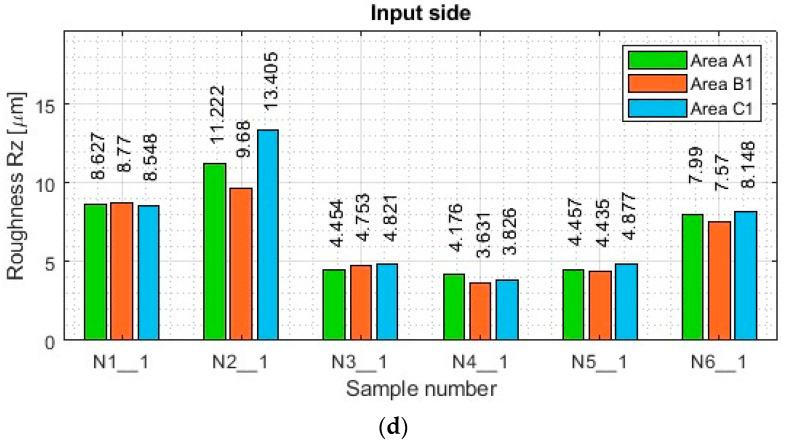
The value of surface topography parameters of nickel alloy samples with thin vertical wall on the input side in areas A1-C1: (**a**) Wa; (**b**) Wz; (**c**) Ra; (**d**) Rz.

**Figure 12:**
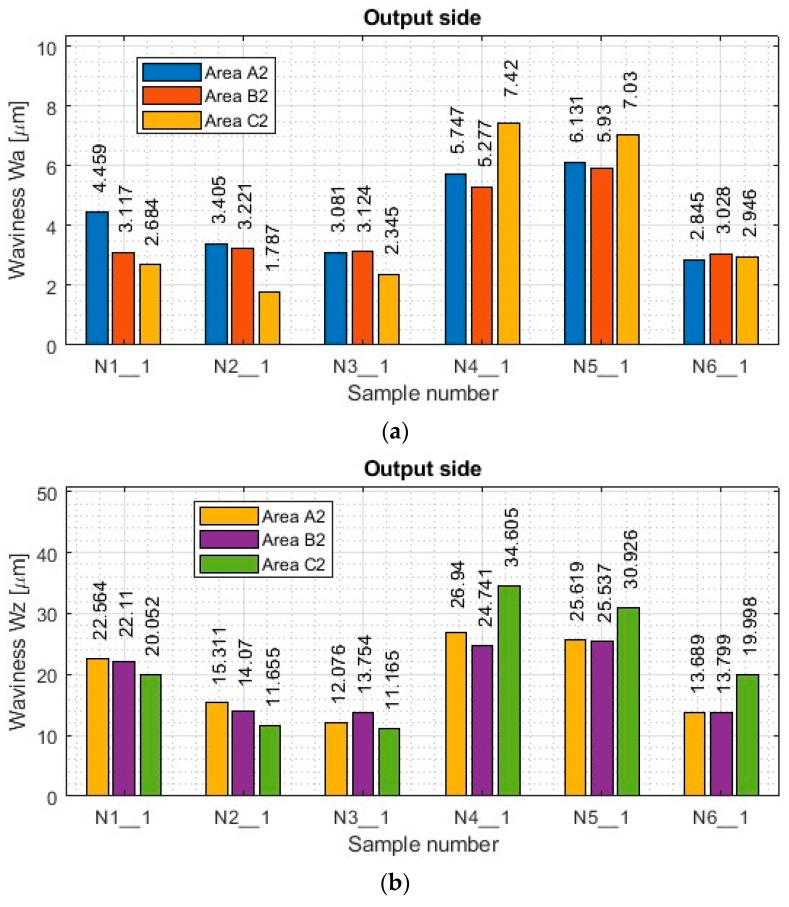

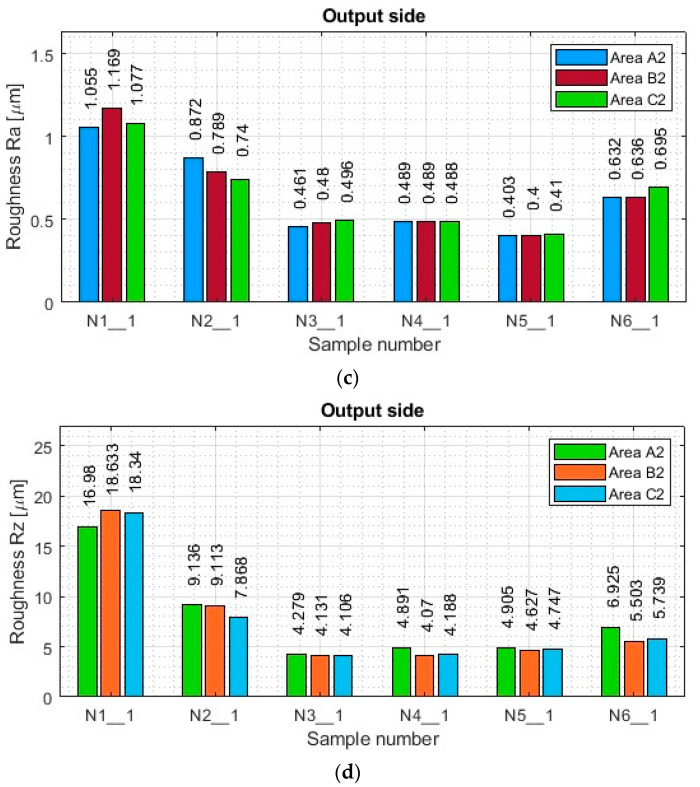
The value of surface topography parameters of nickel alloy samples with thin vertical wall on the output side in areas A2-C2: (**a**) Wa; (**b**) Wz; (**c**) Ra; (**d**) Rz.

A summary of the results of the determined surface topography parameters is included in the Appendix B in Table A1 and Table A2. Based on the data contained therein, histograms of the waviness parameters Wa and Wz and roughness parameters Ra and Rz were prepared for both machined sides. Histograms of selected surface topography parameters are shown in Figure 11 (input side) and Figure 12 (output side).

As for the results of the titanium alloy samples (see [1]), from the data shown in Figure 11 and Figure 12, one notices the similar nature of the plots between the Wa and Wz ripple plots in each measurement area. Even between the machined sides (inputs and outputs), similar obtained ripple values are observed in the corresponding measurement areas.

Focusing on the mutual comparison of the values of the waviness parameters Wa and Wz separately in the range of the lateral face milling strategy and separately in the range of the lateral cylindrical milling strategy, the opposite tendency is apparent for titanium alloy [1], where the smallest values were obtained for the general purpose milling cutter. In contrast, the largest values were obtained for the cutter for high-speed machining. In the case of the nickel alloy samples, the smallest values of ripple parameters were obtained for samples made with the tool for high-speed machining (N3_1 and N6_1), while the largest values were observed for samples made with the tool for general purposes (N1_1 and N4_1). The surface waviness obtained with the tool for high-performance machining using side face milling (N2_1) has similar values to those obtained after machining with the tool for high-speed machining (N3_1) using the same strategy. Sample N5_1 made using cylindrical side milling shows results comparable to those obtained when using the tool for general purposes (N4_1).

For nickel alloy samples, the values of waviness parameters Wa and Wz are also smaller for samples obtained with the side face milling strategy (N1_1–N3_1) compared to side cylindrical milling (N4_1–N6_1). For samples made with the tool for general purposes (N1_1 and N4_1) and the tool for high-performance machining (N2_1 and N5_1), the differences in the Wa and Wz waviness values between the strategies are up to two times (with less for side face milling—N1_1 and N4_1), while for the tool for high-speed machining (N3_1 and N6_1) the results between the strategies are similar to each other—but also with the slight advantage of smaller values for the side face milling strategy (N3_1).

The presented results of the ripple parameters in each of the samples (N1_1–N6_1) show a scattering of values between the measured areas. No dependence was observed in the distribution of the ripple parameters in the measured areas for the samples made by the lateral face milling strategy (N1_1–N3_1). In terms of lateral cylindrical milling (N4_1–N6_1), increases in the waviness parameters Wa and Wz in the C1 and C2 areas are also observed, resulting from the occurrence of a chamfer contained within the measurement area (looks at Figure 3). For this strategy (samples: N4_1–N6_1), the value of the waviness between areas within the range of its sample (between A, B and C) is not repeatable—similar to the case of titanium alloy [1]. However, a similarity in the distribution of the plots between both the Wa and Wz parameters and the machined sides (inputs and outputs) is apparent.

Comparing the nickel alloy samples (N1_1–N6_1) with each other in terms of the obtained Wa and Wz ripple values on both machined sides, it was observed that smaller values of these parameters were obtained on the surface on the input side (compared to the output side). In this case, too, it can be concluded, as in the case of the titanium alloy samples [1], that this is the result of greater wall stiffness for the entry side, in which the thickness of the thin wall is greater by the width of the cut layer in a single pass.

Based on the graphs of roughness Ra and Rz shown in Figure 11 and Figure 12 for the nickel alloy samples, it can be seen that there is a relationship in the distribution of the parameters between the two, which was not the case for the titanium alloy samples [1]. Comparing each other’s graphs of the roughness parameters Ra and Rz for individual samples, it can be seen that they are similar to each other. This means that the distribution of the Rz parameter in the different measurement areas of a given sample is similar to the distribution of the Ra parameter.

For nickel alloy samples made with the tool for general purposes (N1_1 and N4_1) and the tool for high-performance machining (N2_1 and N5_1), there is also the phenomenon of higher waviness values with lower roughness values for lateral face milling (compared to cylindrical) and lower waviness values with higher roughness values for lateral cylindrical milling (compared to face milling). The reason for this phenomenon is analogous to that of titanium alloy samples [1], i.e., for lateral cylindrical milling (N4_1 and N5_1), there is a deflection of the thin wall under the pressure of the milling cutter over the entire height of the wall, which is why higher waviness values are observed. The smaller values of the roughness parameters are because the surface of the thin wall is made in a single pass, so there are no perceptible boundaries between passes. For the samples made with the tool for high-speed machining (N3_1 and N6_1), the distribution of roughness results is analogous to that of waviness, i.e., smaller roughness values were observed for the side face milling strategy (N3_1) than for side cylindrical milling (N6_1). This is the opposite of the trend for the other nickel alloy samples (N1_1, N2_1, N4_1, N5_1). It is assumed that a greater depth of cut and a smaller radial depth should be used during high-speed machining. In this case, the reverse application (less depth of cut and greater radial depth) produced more favorable roughness parameters, which is an interesting observation.

Focusing on the individual areas of each of the nickel alloy samples (N1_1–N6_1), it is observed that roughness Ra and Rz adopt relatively stable values on both the input and output sides for both milling strategies compared to the results presented for the titanium alloy samples [1]. Nickel alloy is characterized by higher hardness and stiffness, which positively affected the values of the measured surface topography parameters in this case. In addition, it is observed that the surface of the nickel alloy samples—thanks to its hardness—did not suffer damage under the influence of thick chips occurring during machining.

The roughness values of Ra and Rz between the input and output sides for samples N3_1–N6_1 are very close to each other, influenced by the high stiffness of the thin wall. For sample N1_1, almost twice the value of the Rz parameter for the input side and a similar value of the Ra parameter (compared to the output side) are observed, while sample N2_1 shows about 20% higher values of the Ra and Rz roughness parameters for the input side (compared to the output side). In terms of roughness values, it is difficult to distinguish the sample with the most favorable surface topography parameters. Therefore, this result will be checked during the statistical analysis of the results.

Summarizing the obtained results of the waviness and roughness parameters during the milling of nickel alloy samples, the same relationship between strategies is observed as in the case of titanium alloy for samples made with the tool for general purposes (N1_1 and N4_1) and the tool for high-performance machining (N2_1 and N5_1). This means that smaller values of waviness parameters and larger values of roughness parameters were measured for lateral face milling (with greater radial depth; sample: N1_1 and N2_1), while larger values of waviness and smaller values of roughness were measured for lateral cylindrical milling (with greater depth of cut; sample: N4_1 and N5_1). An interesting observation is the graphs obtained for the samples made with the tool for high-speed machining (N3_1 and N6_1), where the trend is different. Smaller values of waviness and roughness parameters were obtained for the lateral face milling strategy (N3_1) compared to cylindrical (N6_1).

### 3.3. Statistical Analysis

The following output parameters were used for the statistical analysis, conducted for samples with thin vertical wall:

- Dimensional and shape accuracy—the maximum deviation values (omitting the sign preceding the value) for each of the measured planes (planes 1–6 and 1’–6’) selected in Section 2.2 were assumed; sixs values for each of the machined sides (input and output).
- Surface topography—the values of ripple parameters Wa, Wz and roughness Ra, Rz determined in 6 measurement areas (areas A1–C1 and A2–C2), which were selected in Section 2.3 (three measurement areas for each of the machined sides—the input and output sides), were adopted.

**Figure 13:**
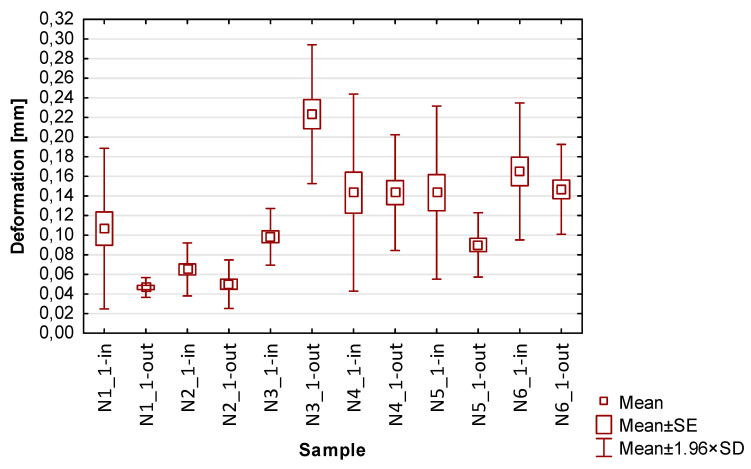
The results of the statistics of the deformation of thin walls.

**Figure 14:**
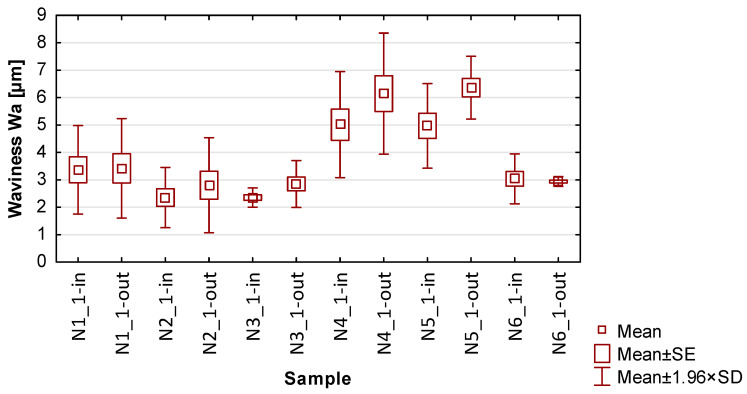
The results of the statistics for waviness Wa.

**Figure 15:**
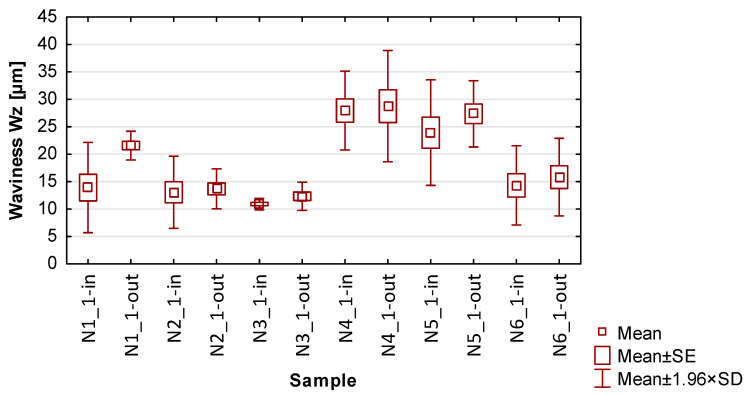
The results of the statistics for waviness Wz.

**Figure 16:**
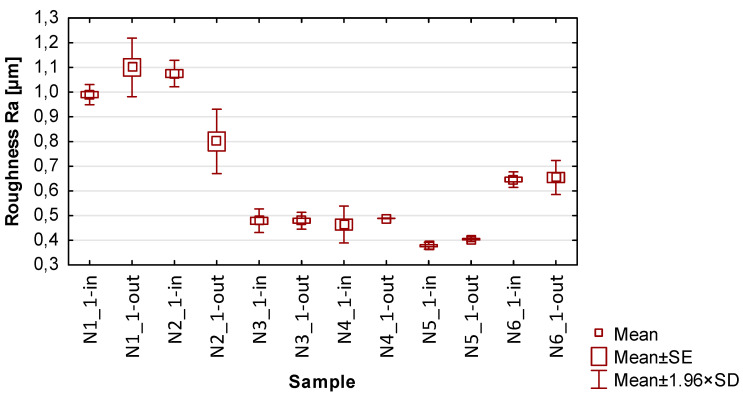
The results of the statistics for roughness Ra.

**Figure 17:**
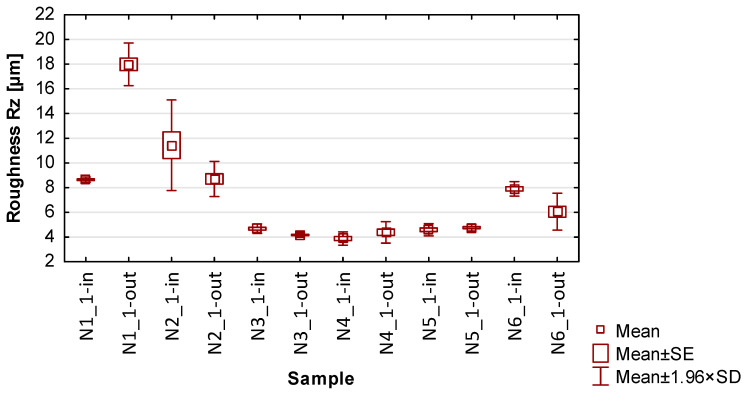
The results of the statistics for roughness Rz.

In the graphs in Figure 13, Figure 14, Figure 15, Figure 16 and Figure 17 showing the basic statistics, the auxiliary designation ‘in’ has been adopted for the input-side plot and ‘out’ for the output-side plot.

Based on the results presented in Table 4 and Figure 13, which contains the results of thin wall deformation for samples N1_1–N6_1, the following observations can be given:

- Focusing on the influence of the cutting tool, it can be seen that the smallest values of average deviations were observed for samples made with the cutter for high-performance machining (N2_1 and N5_1). When considering the other two tools, it can be seen that smaller deviation values were obtained using the tool for general purposes (N1_1 and N4_1) compared to the cutter for high-speed machining (N3_1 and N5_1);
- When using lateral face milling, smaller deviation values were obtained compared to cylindrical milling;
- When lateral cylindrical milling is used, a wider spread of deviation values is observed compared to lateral face milling;
- The smallest deviation values with relatively low scatter were obtained for the sample made by side face milling strategy using the tool for high-performance machining-N2_1;
- The largest values of deviations with a large spread were obtained for sample N3_1. It should be mentioned here that the input side presents two times lower deviations in comparison with the output side;
- In addition to the aforementioned difference between the sides for sample N3_1, for the other samples, smaller average values of deviations on the output side compared to the input side are observed;
- Comparing the deviation result of individual nickel alloy samples with the values obtained in papers [20,21,22,23,24] containing deformation results for titanium alloy samples, it can be seen that lower maximum values were obtained for sample N2_1 (maximum deviation: 0.08 mm). It is worth mentioning that close values of deviations to those obtained in the cited publications are also shown by sample N1_1 (maximum deviation: 0.14).

Based on the results presented in Table 5, Table 6, Table 7 and Table 8 and Figure 14, Figure 15, Figure 16 and Figure 17, containing the results of selected surface topography parameters (waviness Wa, Wz and roughness Ra, Rz) for samples N1_1–N6_1, the following observations can be given:

- Focusing on the influence of the cutting tool, its effect on the measured surface topography parameters is observed in various ways. The selection of a suitable cutting tool should be preceded by the definition of the characteristics of the finished product;
- The use of cylindrical side milling (compared to face milling) provides lower average values and allows for minimizing the scatter of roughness parameter results. In the case of waviness testing, the trend is the opposite, i.e., the use of lateral face milling (compared to cylindrical) gives lower values for waviness parameters;
- A favorable set of surface topography parameters with low scatter was obtained for sample N3_1, made with the tool for high-speed machining along with the use of side face milling. It should be noted, however, that according to the data presented in Table 5 and Figure 13, this sample had the highest values of thin wall deviations;
- The obtained surface topography results show that there is no significant difference in values between the machined sides, i.e., between the input and output sides;
- Analyzing the globally obtained results of surface topography, it is difficult to give additional and more detailed relationships resulting from the distribution of values—the measured values are distributed randomly, with no clear analogies.

In summary, based on the cited statistical results, it is not possible to clearly determine for which case the smallest values of deviations and surface topography parameters were obtained. The assumed conditions affect the measured values in different ways—in some cases, the minimization of selected parameters causes an increase in others. Therefore, the selection of cutting conditions should be preceded by the definition of the expected quality characteristics of the finished product (such as acceptable dimensional deviations and maximum values of roughness and waviness). Such an approach will ensure that a compromise between the characteristics of the finished product is achieved.

## 4. Summary and Conclusions

As part of the experiment, samples were prepared from Inconel 625 nickel alloy containing a thin wall in a vertical orientation. Machining was carried out using a constant material removal rate, as well as cutting speed and feed rate. Three different monolithic cutters, dedicated to different machining methods, were adopted for the study, as well as two strategies that engaged the face and cylindrical parts of the tool to different degrees. The prepared samples allowed the measurement and determination of the deformation of the thin wall in selected sections (in the direction perpendicular and parallel to the bottom of the sample) using a 3D optical scanner, as well as the determination and evaluation of the measured surface topography parameters in the adopted areas on both machined sides.

Based on the results presented, the following observations are noted:

- A distinctive feature was noted on the surface of the samples. The samples contained a chamfer that occurred as the tool exited the material on both machined sides;
- Smaller values of dimensional deviations were observed when machining with a lateral face milling strategy compared to lateral cylindrical milling. It was observed that samples machined using lateral face milling (N1_1–N3_1) are characterized by an approximately constant sample thickness but inclined to one side, while an inverted cone shape appears for samples machined using lateral cylindrical milling (N4_1–N6_1);
- In terms of cutting tool selection, the lowest deviations in terms of strategy were obtained when using the tool for high-speed machining (N2_1 for face side milling and N5_1 for cylindrical side milling);
- The lowest deviation values were obtained for sample N2_1, which was machined using side face milling;
- Despite the use of nickel alloy, which is a harder material compared to titanium alloy, relating the obtained deviation results to the cited publications [20,21,22,23,24] shows that lower values were obtained for sample N2_1 (maximum deviation: 0.08 mm), while for sample N1_1 (maximum deviation: 0.14 mm) the value was similar;
- It was observed that the use of a lateral face milling strategy compared to lateral cylindrical milling yields lower values of the waviness parameters Wa and Wz with higher values of the roughness parameters Ra and Rz;
- Face side milling (compared to cylindrical milling) provides lower and more stable results for waviness parameters, while cylindrical side milling (compared to face side milling) provides lower and more stable results for roughness parameters;
- It is not possible to say unequivocally for which set of input parameters the lowest values of output quantities were obtained. Different effects of input parameters on quality features are observed. Therefore, the selection of cutting conditions should be preceded by the determination of the values of the critical quality characteristics of the machined surface.

Comparing the results of the present study for nickel alloy samples with the data presented in the article [1] for titanium alloy samples (with identical sample shape and the same processing and measurement conditions), it is observed:

- Greater stability in the values of surface topography parameters in the measuring areas was observed when using nickel alloy compared to those obtained for titanium alloy samples;
- The use of titanium alloy, compared to nickel alloy, allowed to obtain smaller values of both measured quantities—the accuracy-dimensional-shape and surface topography parameters comparing the results for samples made under the same machining conditions;
- The machined surface of the nickel alloy samples—thanks to its hardness—did not suffer damage under the influence of coarse chips occurring during machining, as was observed during the milling of titanium alloy samples. As a result, greater stability of the surface topography parameters is observed between the measuring areas.

The authors see possible research directions for the measurement of thin-walled structures:

- Expanding the experiment to include samples containing other shapes of thin-walled structures;
- Conducting tests on samples containing different thin wall thicknesses;
- Using other methods to support the material during milling;
- Experimenting with other material groups;
- Extending the experiment to other cutting parameters to determine a function describing the effect of input parameters on output quantities.

In addition, it should be remembered that the presented scope of research is important in the field of structures, including the aerospace field, but one cannot forget about the equally important issues of subsurface damage, which can be evaluated using microhardness and residual stress analysis, and microstructural changes resulting from heat generated during machining. For parts working under specific conditions, this may have leading significance. The aforementioned properties and characteristics can also be the subject of further research.

## Figures and Tables

**Table 1:** The mechanical properties of nickel alloy Inconel 625 (based on: [59]).

Mechanical Properties	Value	Unit
Tensile strength R_m_	min. 760	MPa
Yield strength 0.2%	min. 380	MPa
Elongation at break	min. 35	[%]
Density	8.44	g/cm^3^

**Table 2:** The chemical composition of nickel alloy Inconel 625 (based on: [52,59]).

Element	Ni	Cr	Mo	Nb	Fe	C	Mn	Si	S	Al	Ti	P	Co
Percentage [%]	≥58	20–23	8–10	3.15–4.15	≤5	≤0.1	≤0.5	≤0.5	≤0.015	≤4.4	≤0.4	≤0.015	≤1

**Table 3:** Summary of selected cutting parameters during the machining of individual samples.

Sample	Tool	Machining Strategy	Depth of Cut a_p_ [mm]	Radial Depth a_e_ [mm]
N1_1	Tool 1	face side milling	2 (8 passes)	4 (1 pass)
N2_1	Tool 2		2 (8 passes)	4 (1 pass)
N3_1	Tool 3		2 (8 passes)	4 (1 pass)
N4_1	Tool 1	cylindrical side milling	16 (1 pass)	0.5 (8 passes)
N5_1	Tool 2		16 (1 pass)	0.5 (8 passes)
N6_1	Tool 3		16 (1 pass)	0.5 (8 passes)

**Table 4:** Summary of the statistical analysis of thin wall deformation.

Parameter	Side	Sample	Mean	Median	Min.	Max.	Var.	Std. Dev.	Std. Error
Thin wall deformation	Input side	N1_1	0.11	0.13	0.04	0.14	0.00	0.04	0.02
		N2_1	0.07	0.07	0.05	0.08	0.00	0.01	0.01
		N3_1	0.10	0.11	0.08	0.11	0.00	0.01	0.01
		N4_1	0.14	0.15	0.07	0.22	0.00	0.05	0.02
		N5_1	0.14	0.14	0.07	0.21	0.00	0.05	0.02
		N6_1	0.17	0.16	0.11	0.22	0.00	0.04	0.01
	Output side	N1_1	0.05	0.05	0.04	0.05	0.00	0.01	0.00
		N2_1	0.05	0.06	0.03	0.06	0.00	0.01	0.01
		N3_1	0.22	0.24	0.16	0.25	0.00	0.04	0.01
		N4_1	0.14	0.14	0.11	0.18	0.00	0.03	0.01
		N5_1	0.09	0.09	0.06	0.11	0.00	0.02	0.01
		N6_1	0.15	0.15	0.11	0.18	0.00	0.02	0.01

**Table 5:** Summary of the statistical analysis of waviness Wa.

Parameter	Side	Sample	Mean	Median	Min.	Max.	Var.	Std. Dev.	Std. Error
Wavines Wa	Input side	N1_1	3.37	3.44	2.51	4.15	0.68	0.82	0.48
		N2_1	2.35	2.46	1.75	2.86	0.31	0.56	0.32
		N3_1	2.36	2.43	2.15	2.49	0.03	0.18	0.10
		N4_1	5.01	4.82	4.14	6.08	0.98	0.99	0.57
		N5_1	4.97	5.03	4.15	5.73	0.62	0.79	0.46
		N6_1	3.04	3.03	2.58	3.51	0.22	0.47	0.27
	Output side	N1_1	3.42	3.12	2.68	4.46	0.86	0.93	0.53
		N2_1	2.80	3.22	1.79	3.41	0.78	0.89	0.51
		N3_1	2.85	3.08	2.35	3.12	0.19	0.44	0.25
		N4_1	6.15	5.75	5.28	7.42	1.27	1.13	0.65
		N5_1	6.36	6.13	5.93	7.03	0.34	0.59	0.34
		N6_1	2.94	2.95	2.85	3.03	0.01	0.09	0.05

**Table 6:** Summary of the statistical analysis of waviness Wz.

Parameter	Side	Sample	Mean	Median	Min.	Max.	Var.	Std. Dev.	Std. Error
Waviness Wz	Input side	N1_1	13.91	15.51	9.15	17.08	17.64	4.20	2.42
		N2_1	13.06	11.51	10.76	16.91	11.24	3.35	1.94
		N3_1	10.91	11.06	10.31	11.34	0.28	0.53	0.31
		N4_1	27.97	27.75	24.41	31.75	13.50	3.67	2.12
		N5_1	23.93	25.42	18.45	27.92	24.06	4.90	2.83
		N6_1	14.31	12.89	11.54	18.50	13.61	3.69	2.13
	Output side	N1_1	21.58	22.11	20.05	22.56	1.79	1.34	0.77
		N2_1	13.68	14.07	11.66	15.31	3.46	1.86	1.07
		N3_1	12.33	12.08	11.17	13.75	1.72	1.31	0.76
		N4_1	28.76	26.94	24.74	34.61	26.81	5.18	2.99
		N5_1	27.36	25.62	25.54	30.93	9.54	3.09	1.78
		N6_1	15.83	13.80	13.69	20.00	13.04	3.61	2.08

**Table 7:** Summary of the statistical analysis of roughness Ra.

Parameter	Side	Sample	Mean	Median	Min.	Max.	Var.	Std. Dev.	Std. Error
Roughness Ra	Input side	N1_1	0.99	0.98	0.98	1.01	0.00	0.02	0.01
		N2_1	1.08	1.07	1.05	1.11	0.00	0.03	0.02
		N3_1	0.48	0.49	0.45	0.50	0.00	0.02	0.01
		N4_1	0.46	0.47	0.42	0.50	0.00	0.04	0.02
		N5_1	0.38	0.38	0.37	0.38	0.00	0.01	0.00
		N6_1	0.65	0.65	0.63	0.66	0.00	0.02	0.01
	Output side	N1_1	1.10	1.08	1.06	1.17	0.00	0.06	0.03
		N2_1	0.80	0.79	0.74	0.87	0.00	0.07	0.04
		N3_1	0.48	0.48	0.46	0.50	0.00	0.02	0.01
		N4_1	0.49	0.49	0.49	0.49	0.00	0.00	0.00
		N5_1	0.40	0.40	0.40	0.41	0.00	0.01	0.00
		N6_1	0.65	0.64	0.63	0.70	0.00	0.04	0.02

**Table 8:** Summary of the statistical analysis of roughness Rz.

Parameter	Side	Sample	Mean	Median	Min.	Max.	Var.	Std. Dev.	Std. Error
Roughness Rz	Input side	N1_1	8.65	8.63	8.55	8.77	0.01	0.11	0.06
		N2_1	11.44	11.22	9.68	13.41	3.50	1.87	1.08
		N3_1	4.68	4.75	4.45	4.82	0.04	0.20	0.11
		N4_1	3.88	3.83	3.63	4.18	0.08	0.28	0.16
		N5_1	4.59	4.46	4.44	4.88	0.06	0.25	0.14
		N6_1	7.90	7.99	7.57	8.15	0.09	0.30	0.17
	Output side	N1_1	17.98	18.34	16.98	18.63	0.78	0.88	0.51
		N2_1	8.71	9.11	7.87	9.14	0.53	0.73	0.42
		N3_1	4.17	4.13	4.11	4.28	0.01	0.09	0.05
		N4_1	4.38	4.19	4.07	4.89	0.20	0.44	0.26
		N5_1	4.76	4.75	4.63	4.91	0.02	0.14	0.08
		N6_1	6.06	5.74	5.50	6.93	0.58	0.76	0.44

## Data Availability

Data are contained within the article.

## Appendix A

Color maps from the report obtained by measuring the vertical deformation of thin-walled samples using a 3D optical scanner are shown in Figure A1, Figure A2, Figure A3, Figure A4, Figure A5, Figure A6, Figure A7, Figure A8, Figure A9, Figure A10, Figure A11 and Figure A12.

## Appendix B

The values of surface waviness and roughness of the vertical thin-wall for samples N1_1–N6_1 are shown in Table A1 and Table A2. Description of the designations in the following tables:

- arithmetic mean waviness—Wa;
- the maximum height of the waviness—Wz;
- skewness of the waviness profile—Wsk;
- the maximum height of peaks of waviness profile—Wp;
- the maximum depth of valleys of waviness profile—Wv;
- the square mean of the waviness deviations—Wq;
- kurtosis of the waviness—Wku;
- the arithmetic mean deviation—Ra;
- the maximum height—Rz;
- skewness of the roughness profile—Rsk;
- the maximum height of peaks of roughness profile—Rp;
- the maximum depth of valleys of roughness profile—Rv;
- the root mean square roughness—Rq;
- kurtosis of the roughness—Rku.

**Table A1:** Parameters of the waviness for the input site measured using a contact profilometer.

Sample Number	Measuring Area	Wa [µm]	Wz [µm]	Wsk	Wp [µm]	Wv [µm]	Wq [µm]	Wku
N1_1	A1	4.15	17.08	1.32	10.19	6.90	4.74	1.91
	B1	3.44	15.51	1.41	10.07	5.44	3.94	2.38
	C1	2.51	9.15	1.20	4.00	5.15	2.74	1.53
	A2	4.46	22.56	1.40	11.17	11.48	5.20	2.21
	B2	3.12	22.11	1.60	15.88	6.23	3.87	3.28
	C2	2.68	20.05	1.75	10.89	9.16	3.50	3.62
N2_1	A1	1.75	11.51	1.56	3.62	7.89	2.09	3.13
	B1	2.46	10.76	1.25	4.87	5.89	2.73	1.69
	C1	2.86	16.91	1.33	5.56	11.35	3.24	2.08
	A2	3.41	15.31	1.36	7.56	7.75	4.06	1.98
	B2	3.22	14.07	1.32	7.81	6.26	3.78	1.88
	C2	1.79	11.66	1.68	4.94	6.71	2.36	3.19
N3_1	A1	2.15	10.31	1.37	5.78	4.54	2.53	2.05
	B1	2.43	11.06	1.36	6.03	5.03	2.83	2.01
	C1	2.49	11.34	1.38	6.20	5.14	2.94	2.07
	A2	3.08	12.08	1.23	5.95	6.13	3.41	1.62
	B2	3.12	13.75	1.24	7.49	6.26	3.48	1.67
	C2	2.35	11.17	1.32	5.84	5.33	2.70	1.90
N4_1	A1	4.82	27.75	1.58	14.58	13.17	6.12	2.75
	B1	4.14	24.41	1.59	12.12	12.29	5.26	2.81
	C1	6.08	31.75	1.50	18.51	13.23	7.47	2.53
	A2	5.75	26.94	1.45	14.78	12.16	6.88	2.43
	B2	5.28	24.74	1.45	13.54	11.20	6.32	2.36
	C2	7.42	34.61	1.31	20.67	13.94	8.31	1.87
N5_1	A1	4.15	18.45	1.42	10.98	7.66	4.98	2.23
	B1	5.03	25.42	1.50	13.25	12.17	6.20	2.50
	C1	5.73	27.92	1.46	14.86	13.06	6.94	2.36
	A2	6.13	25.62	1.38	14.53	11.09	7.26	2.06
	B2	5.93	25.54	1.39	14.13	11.40	7.05	2.11
	C2	7.03	30.93	1.35	16.49	14.44	8.26	1.99
N6_1	A1	2.58	11.54	1.24	6.57	4.97	2.88	1.66
	B1	3.03	12.89	1.23	7.42	5.47	3.35	1.62
	C1	3.51	18.50	1.32	11.77	6.73	4.02	1.91
	A2	2.85	13.69	1.28	7.76	5.93	3.22	1.80
	B2	3.03	13.80	1.29	7.85	5.95	3.44	1.83
	C2	2.95	20.00	1.51	13.09	6.91	3.61	2.64

**Table A2:** Parameters of the roughness for the input site measured using a contact profilometer.

Sample Number	Measuring Area	Ra [µm]	Rz [µm]	Rsk	Rp [µm]	Rv [µm]	Rq [µm]	Rku
N1_1	A1	0.98	8.63	1.62	4.90	3.72	1.23	3.11
	B1	1.01	8.77	1.60	4.63	4.14	1.26	3.03
	C1	0.98	8.55	1.61	4.51	4.04	1.23	3.04
	A2	1.06	16.98	2.00	10.73	6.25	1.41	5.62
	B2	1.17	18.63	2.39	10.40	8.23	1.65	8.16
	C2	1.08	18.34	2.23	9.25	9.09	1.50	6.98
N2_1	A1	1.07	11.22	1.82	4.83	6.40	1.43	3.99
	B1	1.05	9.68	1.81	5.26	4.42	1.41	3.86
	C1	1.11	13.41	1.87	6.85	6.56	1.47	4.50
	A2	0.87	9.14	1.96	5.10	4.03	1.21	4.69
	B2	0.79	9.11	2.01	5.08	4.03	1.10	5.01
	C2	0.74	7.87	1.91	3.57	4.30	1.01	4.48
N3_1	A1	0.45	4.45	2.04	2.67	1.79	0.64	5.08
	B1	0.49	4.75	1.95	2.74	2.01	0.69	4.54
	C1	0.50	4.82	1.90	3.05	1.78	0.68	4.36
	A2	0.46	4.28	1.70	2.44	1.84	0.59	3.56
	B2	0.48	4.13	1.62	2.21	1.92	0.60	3.10
	C2	0.50	4.11	1.63	2.27	1.83	0.63	3.14
N4_1	A1	0.47	4.18	1.55	2.06	2.12	0.58	2.76
	B1	0.42	3.63	1.60	1.72	1.91	0.53	2.98
	C1	0.50	3.83	1.52	1.88	1.95	0.62	2.66
	A2	0.49	4.89	1.58	2.74	2.16	0.61	2.93
	B2	0.49	4.07	1.53	2.15	1.92	0.60	2.70
	C2	0.49	4.19	1.55	2.28	1.91	0.60	2.79
N5_1	A1	0.38	4.46	1.83	2.59	1.96	0.49	4.38
	B1	0.37	4.44	1.79	2.57	1.87	0.48	4.09
	C1	0.38	4.88	1.90	3.01	1.87	0.51	4.86
	A2	0.40	4.91	1.95	3.07	1.84	0.54	5.11
	B2	0.40	4.63	1.83	2.88	1.75	0.52	4.35
	C2	0.41	4.75	1.86	2.96	1.79	0.54	4.48
N6_1	A1	0.65	7.99	1.99	5.16	2.83	0.87	5.36
	B1	0.63	7.57	1.84	5.16	2.41	0.82	4.47
	C1	0.66	8.15	1.90	5.01	3.13	0.87	4.93
	A2	0.63	6.93	1.84	4.42	2.51	0.83	4.44
	B2	0.64	5.50	1.60	3.18	2.33	0.79	3.06
	C2	0.70	5.74	1.58	3.28	2.46	0.86	2.96
